# Neurobehavioral Interpersonal Synchrony in Early Development: The Role of Interactional Rhythms

**DOI:** 10.3389/fpsyg.2019.02078

**Published:** 2019-09-18

**Authors:** Gabriela Markova, Trinh Nguyen, Stefanie Hoehl

**Affiliations:** Department of Applied Psychology: Health, Development, Enhancement and Intervention, Faculty of Psychology, University of Vienna, Vienna, Austria

**Keywords:** interpersonal synchrony, entrainment, social interactions, early development, rhythms

## Abstract

Social interactions are essential for understanding others’ actions and their mental and affective states. Specifically, interpersonal coordination – also referred to as synchrony – allows actors to adjust their behaviors to one another and thus demonstrate their connectedness to each other. Much behavioral research has demonstrated the primacy of mutually synchronized social exchanges in early development. Additionally, new methodological advances now allow us to examine interpersonal synchrony not only at the behavioral and physiological but also neural level. Nevertheless, it remains unclear how infants and their caregivers actually achieve interpersonal synchrony in their exchanges. Here we discuss recent evidence showing that adults provide rhythmical information during early social interactions with their infants, such as affective touch and singing. We propose that entrainment to these social rhythms underlies the formation of interpersonal synchrony and thus stimulates reciprocal interactions between infants and their caregivers.

Interactions with others are essential for virtually all areas of human development. It is a vital question how very young infants begin to make sense of others in order to learn from them. Far from being passive observers, infants are embodied agents in their interactions with other people, engaging with them in mutual dynamic exchanges (De Jaegher and Di Paolo, 2007; Schilbach et al., 2013). These exchanges are essential for infants’ developing understanding of self and others. For instance, through the caregiver’s mirroring of the infant’s emotional expressions, the infant may learn to associate affective experiences with facial expressions (Keysers and Perrett, 2004). Coordination of this kind is often referred to as (interpersonal) synchrony, a “dynamic process by which hormonal, physiological, and behavioral cues are exchanged” and reciprocally adjusted between two or more persons (Feldman, 2012). Much research shows that interpersonal synchrony is a defining characteristic of early infant-caregiver interactions and can be measured not only at the behavioral but also physiological (e.g., oxytocin, cardiac output) and possibly neural level (see Feldman, 2007, for review). We distinguish between concurrent synchrony (e.g., joint action, mutual gaze, mirroring) and sequential synchrony (e.g., turn-taking, reciprocity, imitation; Feldman, 2007). Moreover, we distinguish between dyadic and triadic forms of synchrony that differ in whether synchrony is achieved within the dyad (i.e., through speech or affect) or whether it is triggered by external stimuli (i.e., music, toys, etc.; see Figure 1), although both ways of achieving synchrony are not mutually exclusive and might sometimes work in combination. Due to the multifaceted characteristics of early interpersonal synchrony, it remains largely unclear how infants and their caregivers synchronize.

**Figure 1:**
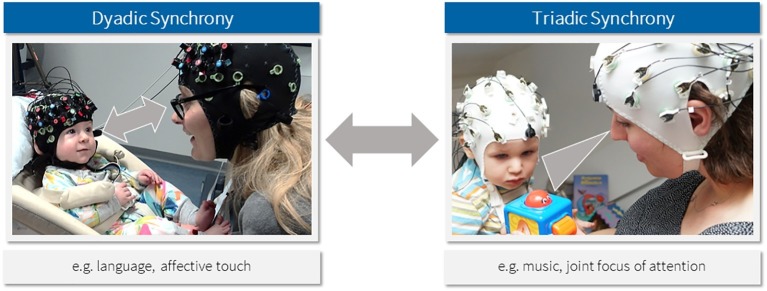
Forms of interpersonal synchrony in caregiver-child interactions.

In this article, we review evidence showing the infant’s complex capacity to interact in a rhythmical social dialogue with others and discuss new methodological approaches that allow the examination of neural synchrony in live interactions. We then describe how rhythmical information that adults provide during social exchanges with their infants, such as affective touch and singing, enhances infant attention and thus stimulates interactions. We conclude by presenting the hypothesis that entrainment to these social rhythms promotes interpersonal synchrony.

## Early Social Interactions

From early on in life, infants recognize and create structures in their engagements with others. Social interactions between infants and their caregivers are mutually regulated systems that are characterized by face-to-face exchanges, close physical contact, and a turn-taking structure, where social engagement and disengagement alternate (Brazelton et al., 1974; Field, 1978; Tronick, 1989; Trevarthen, 1993; Papousek, 1995). Even neonates sequentially synchronize their behaviors with those of adults (Condon and Sander, 1974), although there seem to be some culturally variable factors influencing early interpersonal synchrony (Gratier, 2003).

Starting at birth infants, are involved in particular routines with caregivers, such as having a nappy changed or being fed. These routines are dynamic and require coordination between the behaviors of the adult and infant. For example, previous research showed that already at 2 months, infants recognize the regularities of an incipient pick-up action, and adjust to them in anticipation to facilitate the coordination of actions with the adult (e.g., increasing body rigidity, or widening the arms to create more space for the mother to hold the infants; Reddy et al., 2013). Thus, participating in these routines may increase infants’ sensitivity to caregivers’ interpersonal actions.

Interpersonal synchrony has provided a framework for studying early human relationships and a growing body of research suggests that it has a biological basis (see Feldman, 2007, 2012, for a review). Interestingly, recent research with adults using simultaneous recordings of brain activities from several persons (i.e., hyperscanning) suggests that concurrent as well as sequential interpersonal synchrony is associated with neural synchronization (e.g., Dumas et al., 2012; Jiang et al., 2012; Konvalinka and Roepstorff, 2012). Critically, studying brain activities during dynamic live interactions provides new insights into the mechanisms of interpersonal synchrony (Hasson et al., 2012).

## How do Brain Activities of Interacting Persons Synchronize?

Interpersonal neural synchronization has been proposed as a fundamental mechanism facilitating interpersonal transmission of information through verbal and non-verbal communication (Dumas et al., 2011; Hasson et al., 2012). In fact, a growing body of evidence suggests that interpersonal neural synchrony facilitates communication and affective co-regulation in adults (Schippers et al., 2010; Hasson et al., 2012; Goldstein et al., 2018). In the behavioral domain, effective interpersonal transmission of information has been linked to both sequential (Wilson and Wilson, 2005) and concurrent behavioral synchrony (Chang et al., 2017, 2019).

We propose that interpersonal synchrony of brain activities is achieved within the dyad through communicative rhythms as much as it may be induced through external rhythms (see Figure 1). In the case of dyadic synchrony, synchronization of neural oscillations may reflect mutual attunement through communicative rhythms (Hasson et al., 2012) that are transmitted interpersonally through the environment by coupling the sensory system of one person to the motor system of another person. Entrainment of internal neuronal oscillations to external rhythms enables optimal processing of rhythmic stimuli, because sensory input can then be sampled during phases of high neuronal excitability (Calderone et al., 2014). Similar to a radio tuned to a specific frequency band, we are thus able to track and predict incoming information (Large and Jones, 1999), e.g., the voice of a communicative partner in a noisy environment.

To investigate which conditions would encourage neural synchronization, Fishburn et al. (2018) measured interpersonal neural synchronization through fNIRS in an active social interaction and compared it to passive observation of the interaction and movie watching. Only active social interaction led to increased interpersonal neural synchronization. Thus, neural synchronization may support social information exchange beyond shared visual stimulation. Further studies have started to look into socio-behavioral outcomes of interpersonal neural synchronization. In the language domain, neural synchrony and entrainment of endogenous brain rhythms to language were found to facilitate verbal communication between conversing adults (Giraud and Poeppel, 2012; Luo and Poeppel, 2012; Zion Golumbic et al., 2013; Dai et al., 2018). Additionally, interpersonal coordination of brain activity has been associated with enhanced gestural communication using fMRI (Schippers et al., 2010) and spontaneous imitation of hand gestures using EEG (Dumas et al., 2010). Thus, coordination of neural activity is not only achieved through language but also involves motor regions. As a third pathway to dyadic coordination, interpersonal affiliation and regulation of affect has been associated with interpersonal synchronization of brain activity mediated through affective touch (Goldstein et al., 2018).

Based on existing behavioral evidence from developmental research as reviewed above and this recent neuroscience research with adults, it stands to argue that interpersonal synchronization of brain rhythms may play a substantive role for caregiver-child coordination, communication, and attachment formation (see also Atzil and Gendron, 2017). However, empirical evidence is still scarce, partly due to the methodological challenges associated with acquiring neurophysiological data from infants and small children (Hoehl and Wahl, 2012). This is augmented by the fact that the infant EEG is dominated by slower frequency rhythms compared to the adult EEG (Thierry, 2005), which could be detrimental to the mutual coordination of internal oscillators between infants and adults. Given that functionally equivalent oscillatory responses peak at lower frequencies in infants than in adults (e.g., alpha peaks at 5–9 Hz in infants compared to 8–12 Hz in adults) (Stroganova et al., 1999), the application of cross-frequency coupling analysis methods might prove useful in the future (Canolty and Knight, 2010; Aru et al., 2015). It is conceivable that slower frequency activity in infants coordinates with higher frequency activity in adults, but to the best of our knowledge this has not yet been shown empirically.

First evidence for adult-infant interpersonal coupling of brain activities comes from a dual-EEG study by Leong et al. (2017), where an adult experimenter sang nursery rhymes to 8-month-old infants either while maintaining eye contact or while looking away from the infant. The authors determined mutual influences on the brain activities of the interaction partners using partial directed coherence, a frequency domain approach to describe the relation between two multivariate time series. Direct gaze enhanced mutual influences of the adults’ and infants’ neural oscillations in the infant alpha band (6–9 Hz). Leong et al. (2017) suggested that eye contact may induce a phase-reset in both interaction partners, consequently facilitating interpersonal neural synchrony, although this hypothesis has yet to be tested directly.

Given that the EEG signal is prone to artifacts caused by eye and body movements as well as speech, fNIRS can be considered a promising additional method to assess interpersonal synchronization of brain activity across different age groups. Yet, similar to fMRI, fNIRS measures brain activation based on oxygenation changes in the blood (Lloyd-Fox et al., 2010), the hemodynamic delay poses as a shortcoming to fNIRS measurements, which are more suitable to capture slower processes than gaze, such as touch and affect (e.g., Pirazzoli et al., 2018). In contrast to dual-fMRI, however, dual-fNIRS allows for testing live face-to-face settings, thus providing a more ecologically valid picture of social interactions. For example, Reindl et al. (2018) had 5- to 9-year-old children play a cooperative or competitive version of a simple button-press game either with their parent or with an experimenter. During cooperation, parents’ and children’s brain activities synchronized in the dorsolateral prefrontal cortex and frontopolar cortex. Interpersonal neural coherence predicted dyads’ cooperative performance in subsequent trials. No significant neural synchrony was observed for parent-child competition, stranger-child cooperation, and stranger-child competition, indicating that interpersonal neural synchrony depends on the relationship to the interactive partner and the task context.

Nguyen et al. (under review) recently extended this research to a natural and dynamic task context asking mothers and their preschool-aged children to perform a puzzle task either in cooperation or individually. Dyads displayed higher interpersonal neural synchrony in frontal and temporo-parietal regions associated with perspective-taking and social-decision making during cooperation compared to individual performance and rest. Synchrony during cooperation was positively associated with dyadic task performance (i.e., number of puzzles solved). Crucially, the natural task allowed for assessing variances in dyadic interactive behavior. Higher behavioral reciprocity as well as child agency were associated with increased neural synchrony. Taken together, these results indicate that interpersonal neural synchrony might be a useful marker for mutual engagement in dynamic social interactions that depends on both partners being responsive and attentive to each other.

Given these first promising findings, hyperscanning between infants, children, and adults could be used in future research to assess both the preconditions and the consequences of interpersonal neural synchrony across development (Hoehl and Markova, 2018). However, it currently remains unclear whether neural synchrony in these tasks really depends on both persons coordinating mentally, behaviorally, and neurally with each other. It is also possible that increased task engagement induces more similar sensory input and, consequently, more similar patterns of brain activity in both partners independently of each other. To our knowledge, only one study has established a causal link between neural and behavioral synchrony through the simultaneous use of transcranial alternating current stimulation (tACS) and assessment of increased concurrent behavioral synchrony between adult participants in a tapping task (Novembre et al., 2017). Ideally, hyperscanning methods can be applied to deepen our understanding of the mechanisms of caregiver-child interactions and mutual attunement. Particularly, the role of perceivable dyadic and triadic rhythms for interpersonal neural synchrony remains to be addressed in future research. Inherently dyadic communicative rhythms (e.g., speech) as well as external rhythms (e.g., music) may be mechanisms enabling interpersonal neural coupling, and consequently interpersonal behavioral synchrony (Hasson et al., 2012; Schirmer et al., 2016). A continuing question remains to what degree these rhythms have to be consciously attended to in the dyadic interaction to be effective and whether transient background rhythms can serve a similar function in facilitating interpersonal synchrony or not. In the next section, we discuss interpersonal rhythms that might bear a particular relevance in early caregiver-child interactions, namely affective touch and singing.

## Rhythms of Early Interactions

Communicative rhythms, such as those introduced through rhythmic touch or singing, are often intuitively used by caregivers to establish interpersonal synchrony with their infants and to down-regulate negative affect (Provasi et al., 2014). Affective touch facilitates the transmission of affective states and has been related to infants’ down-regulation of distress as well as children’s emotional development (Hertenstein et al., 2006; Feldman et al., 2011). In human adults, gentle stroking has been shown to activate C-tactile fibers (Löken et al., 2009) that preferentially respond to stroking with medium-velocity, which is associated with pleasant touch and affiliative bonding. The affiliative nature of touch is highlighted by research showing that hand-holding in adults increases both physiological synchrony and interpersonal neural synchronization in brain networks associated with analgesia (Goldstein et al., 2017, 2018).

Compared to studies with adults, there is relatively little research on the neurophysiological mechanism of affective touch in caregiver-child interactions. Similar to adults, gentle stroking elicits specific behavioral, physiological, and neural responses infants (Fairhurst et al., 2014; Jönsson et al., 2018; Pirazzoli et al., 2018). In addition, infants are able to adjust their cardiorespiratory patterns to their mother when passively lying on her body (Van Puyvelde et al., 2015). It seems plausible that infants might be able to recognize stroking and cardiorespiratory patterns during skin-to-skin contact with their caregiver as a form of interpersonal rhythm (Provasi et al., 2014). Rhythmic stroking and close physical proximity may thus allow the dyad to attune to and, consequently, coordinate with each other.

Infant-directed (ID) singing may be another way for caregivers to introduce rhythms into their interactions with infants (see e.g., Cirelli et al., 2018, for review). Parents may use ID singing to direct infants’ attention to them, regulate their affective states, and teach them about auditory patterns (Trainor, 1996). At the same time, playful ID singing can be used to activate and engage the infant in interactive games (Rock et al., 1999; Cirelli et al., 2019). Overall, engagement and connectedness within musical experiences may facilitate social connections between infants and their parents (Fancourt and Perkins, 2017; Cirelli et al., 2018). Thus, we propose that there is another potential function of ID signing, namely, to establish interpersonal synchrony.

Behavioral research shows that very young infants are sensitive to the temporal organization of musical sequences. For example, newborn infants show sensitivity to onsets, offsets, and tempo of tone sequences (Háden et al., 2015), and respond to omissions of metrically important tones in a rhythmic pattern (Winkler et al., 2009). Five-month-olds move rhythmically to periodic auditory patterns (Zentner and Eerola, 2010), and 7- and 15-month-olds show neural entrainment to the beat and meter of rhythmic sounds (Cirelli et al., 2016). ID singing is characterized by an enhanced regularity of its canonical temporal organization due to its metrical structure (Nakata and Trehub, 2004, 2011). Newborns are more attentive to ID than to non-ID singing, and 5- to 6-month-olds are more engaged when listening to singing than to speech (see Provasi et al., 2014, for a review). Though this has yet to be established empirically, we assume that ID singing may induce greater neural and physiological entrainment to rhythmic patterns in infants than speech or adult-directed singing. In particular, infants’ rhythmic brain activity (and perhaps heart rate and respiration) may entrain to their mothers’ singing, that is, become phase-locked to the rhythms carried through the mothers’ voice.

## Conclusions and Future Directions

In this review, we have emphasized the potential of simultaneous measurement of brain activities in dynamic live interactions to unravel the underpinnings of interpersonal synchronization in caregiver-child interactions. The presented evidence highlights the role of bio-behavioral entrainment to communicative rhythms, conveyed through gaze, affective touch, and singing, as a potential mechanism to enable interpersonal synchronization of brain activity and, in consequence, behavior.

To deepen our understanding of interpersonal synchronization, we propose that it is indispensable to directly examine entrainment to social rhythms that occur spontaneously in naturalistic contexts, including joint attention, play, speech, and daily routines. Adults generally employ a manifold of rhythms: singing to an infant may be accompanied by synchronous rocking or bouncing, just as affective touch may be accompanied by calming vocalizations. The synchrony of sensory input across modalities likely facilitates entrainment, as infants attune to the envelope of several rhythmic stimuli. Accordingly, we have to consider the interplay of different modalities when examining interpersonal entrainment (see also Phillips-Silver et al., 2010).

Both short- and long-term implications of neurobehavioral synchronization are still to be determined. Based on existing evidence, short-term outcomes of neurobehavioral synchrony are enhanced social connectedness, effective communication as well as interpersonal regulation (Feldman, 2007; Stephens et al., 2010; Leong et al., 2017). Because these outcomes pose excellent conditions for social learning (Hoehl and Markova, 2018), future research should examine the effects of interactional rhythms and interpersonal synchrony on communication and learning in early development.

In the long term, neurobehavioral synchronization has been linked to the development of social competence, secure attachment and bonding (Atzil and Gendron, 2017). Longitudinal studies examining the development of neurobehavioral synchronization can provide insights on how interpersonal attunement might benefit a child’s development and also under what circumstances (too much) synchrony may be detrimental. In fact, only about 30% of child-caregiver interactions are characterized by synchrony, while miscoordinations and repairs thereof seem beneficial for children’s developing self-efficacy (e.g., Tronick, 2014). Thus, too much synchrony may be indicative of intrusive parenting practices (Beebe et al., 2010).

In this paper, we have stressed the importance of dyadic and triadic social rhythms as critical factors that may co-determine interpersonal synchrony and thus yield a deeper understanding of its multimodal organization, its role in early engagements, and its developmental outcomes. It remains a goal for future research to study the mechanisms and functions of interpersonal rhythms at the behavioral, physiological, and neural level and examine how they relate to each other in early caregiver-child interactions.

## Author Contributions

GM, TN, and SH jointly contributed to the conceptualization and writing of this article.

### Conflict of Interest Statement

The authors declare that the research was conducted in the absence of any commercial or financial relationships that could be construed as a potential conflict of interest.
